# Security-Related Hardware Cost Optimization for CAN FD-Based Automotive Cyber-Physical Systems

**DOI:** 10.3390/s21206807

**Published:** 2021-10-13

**Authors:** Yong Xie, Yili Guo, Sheng Yang, Jian Zhou, Xiaobai Chen

**Affiliations:** 1 School of Computer Science, Nanjing University of Posts and Telecommunications, Nanjing 210023, China; zhoujian@njupt.edu.cn (J.Z.); chenxb86@njupt.edu.cn (X.C.); 2 School of Computer and Information Engineering, Xiamen University of Technology, Xiamen 361024, China; 1922032011@stu.xmut.edu.cn (Y.G.); 1722011058@stu.xmut.edu.cn (S.Y.)

**Keywords:** automotive cyber-physical systems, hardware cost, design space exploration algorithm, cyber security, CAN FD

## Abstract

The introduction of various networks into automotive cyber-physical systems (ACPS) brings great challenges on security protection of ACPS functions, the auto industry recommends to adopt the hardware security module (HSM)-based multicore ECU to secure in-vehicle networks while meeting the delay constraint. However, this approach incurs significant hardware cost. Consequently, this paper aims to reduce security enhancing-related hardware cost by proposing two efficient design space exploration (DSE) algorithms, namely, stepwise decreasing-based heuristic algorithm (SDH) and interference balancing-based heuristic algorithm (IBH), which explore the task assignment, task scheduling, and message scheduling to minimize the number of required HSMs. Experiments on both synthetical and real data sets show that the proposed SDH and IBH are superior than state-of-the-art algorithm, and the advantage of SDH and IBH becomes more obvious as the increase about the percentage of security-critical tasks. For synthetic data sets, the hardware cost can be reduced by 61.4% and 45.6% averagely for IBH and SDH, respectively; for real data sets, the hardware cost can be reduced by 64.3% and 54.4% on average for IBH and SDH, respectively. Furthermore, IBH is better than SDH in most cases, and the runtime of IBH is two or three orders of magnitude smaller than SDH and state-of-the-art algorithm.

## 1. Introduction

### 1.1. Background and Motivations

As security is not considered in in-vehicle networks’ specification, the employment of various network interfaces (wireless or wired) in automobiles poses great cyber-security challenges to the safety of automotive cyber-physical systems (ACPS). For example, CAN is the most widely employed in-vehicle network in automobiles, but it is vulnerable to replay attack, masquerade attack, and DoS attack [1]. CAN with flexible data-rate (CAN FD) is proposed in 2011 and viewed as the next generation of CAN technology, which can elevate the bandwidth of the transmission phase to 8 Mbps, but the security vulnerabilities of CAN are not resolved [2]. Many kinds of cyber attacks have been identified in in-vehicle networks such as CAN, CAN FD, and FlexRay, and the potential security vulnerabilities have lead to car recall event [3]. As a result, automotive stakeholders are trying to employ different kinds of security enhancing mechanisms to safeguard in-vehicle networks, such as adding message authentication code (MAC) into message for integrity and availability [4,5] (recommended and specified in AUTOSAR SecOS specification [6]), message encryption for confidentiality [7], intrusion detection, and message ID obfuscation [8]. The authors of [9] give an extensive survey about the security vulnerabilities and proposed security protection mechanisms of in-vehicle networks. However, as it is too time- and memory-consuming to implement the cryptographic algorithms in software, processors with a dedicated security coprocessor such as hardware security module (HSM) are recommended by the auto industry to be used in the new generation of ECUs [10]. Main automotive chip makers like Infineon and Renesas are already producing multicore processors with HSM, such as Infineon Aurix and Renesas R-Car. The HSM-based security-enhancing approach can reduce the delay overhead to an acceptable level. For example, as the real implementation on Aurix [11] shows that software-based implementation of AES128 consumes 1209 μs, while the HSM-based implementation only needs 62 μs. HSM offer several advantages, such as cryptographic engine (hardware implementation of symmetric/asymmetric cryptographic algorithms and hash functions, such as AES128, ECC256, SHA256, and others), secure key storage, secure log, and others, which would allows the ECU’s host core to devote its full power to the other tasks, and offers ECU manufacturers and automakers a powerful plug-and-play security solution that can be easily adapted to their own security requirements. World-renown automotive suppliers are providing HSM-based security solutions that combines Aurix and HSM, such as ESCRYPT’s CycurHSM and Elektrobit’s zentur HSM.

Although HSM frees the host core from computing-intensive security tasks, it brings considerable hardware cost. Taking Aurix processores from Infineon as an example, the Aurix TC299TP128F300N and Aurix TC299T128F300S have similar performance (in terms of CPU and memory), but the price of Aurix TC299TP128F300N (about 47 dollars) is higher than that of the Aurix TC299T128F300S (about 41 dollars) as it has the HSM, thus the adding of HSM increases the hardware cost by about 15% [12]. However, given that automobiles are mass-produced consumer products which are very cost sensitive, it is not cost-efficient to add HSM in all ECUs. According to the authors of [13], Toyota and Honda only integrate HSM-based security protection mechanisms into a few ECUs implementing safety-critical functions, such as engine ECU, brake ECU, and steering ECU. As a result, it is important to reduce the HSM introduced hardware cost by minimizing the number of ECUs equipped with HSM. In this paper, we formulate an optimization problem to minimize the number of HSMs required for a given ACPS, where both the task mapping, task scheduling and message scheduling are explored subject to both deadline and security constraints.

### 1.2. Contributions

In this paper, we observe that HSM-based security protection mechanisms introduce considerable hardware cost, which poses great challenges to cost management of car manufacturers. Thus, we formulate a new design space exploration (DSE) problem to reduce the hardware cost for security-enhanced ACPS. To the best of our knowledge, this is the first time that HSM introduced hardware cost is integrated into the DSE of CAN FD-based ACPS. The main contributions of this paper are as follows. (1) We provide a stepwise decreasing-based heuristic DSE algorithm (SDH) which reduces the design space into smaller one based on the decreasing number of HSMs. (2) As the performance of SDH deteriorates as the searching space expands, we provide another interference-balancing based heuristic algorithm (IBH) which can get an equal or even better result with much shorter runtime by comparing with SDH. (3) By comparing with the state-of-the-art algorithm based on both synthetic and real data sets, the efficiency of the proposed algorithms is verified.

This paper is organized as follows. Section 2 surveys the related work. Section 3 presents the system models and key assumptions. In Section 4, the details about the SDH and IBH algorithms are given for the hardware cost optimization problem. Section 5 presents the experimental results, and the paper is concluded in Section 6.

## 2. Related Work

Cost optimization is one of the key objectives for DSE of embedded systems. In [14], the authors first transfer reliability goal of the application to that of each task, and then resource cost is minimized by heuristically assigning tasks to the processors. In [15,16], the authors propose to minimize the development cost of embedded systems with genetic algorithm-based and tabu search-based heuristics, respectively. In [17], the authors propose a two-stage solution for function safety risk assessment and development cost optimization of software-defined vehicles, where both the reliability and real-time requirements are considered. However, the above-mentioned works consider resource cost and development cost, and they employ different cost models. Hardware cost is an essential part of the cost of embedded systems, especially for mass-produced ACPS. In [18], the authors present two heuristic algorithms to minimize hardware cost for parallel embedded systems, where both the functional safety and real-time requirements are considered. In [19], the authors combine genetic algorithm and simulated annealing to reduce the hardware cost and energy consumption of embedded products while satisfying the hard real-time and reliability requirements of safety-critical applications. In [20], the authors present three price performance-driven heuristic algorithms for hardware cost minimization of embedded systems, which consider both real-time and reliability requirement. In [21], the authors present a multi-population genetic algorithm towards optimizing both operation time and the number of required processing units for distributed real-time systems. In [22], the authors try to reduce the hardware cost by minimize the number of required processors to schedule an application, where considerably memory requirements and application latency are reduced by comparing with related approaches while meeting the same throughput constraint. In [23], the authors shows how to minimize the number of required processors for feasible running the parallel real-time tasks. However, our work considers to minimize security-enhancing related hardware cost of ACPS by minimizing the number of HSMs, where both security and real-time requirements are considered. In [24], the authors try to minimize the hardware cost for security-aware ACPS, but it considers the FlexRay as the in-vehicle network. Our work considers the CAN FD-based ACPS, and we assume a more general system model with two security levels.

## 3. System Models and Key Assumptions

### 3.1. System Model

**Figure 1:**
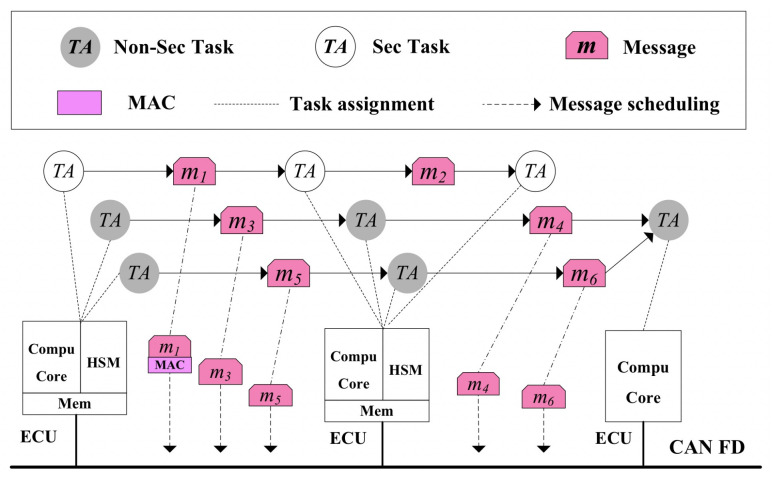
DSE for CAN FD-based ACPS.

Figure 1 shows a typical safety-critical electronic subsystem inside the ACPS, where several ECUs are interconnected by CAN FD. We assume that there is a task set in each ECU and tasks are scheduled with static priority-based preemptive scheduling algorithm according to the AUTOSAR specification [25]. Messages are transmitted on the bus to realize the communication and cooperation between communicating tasks which are assigned to different ECUs. ECU set is represented as $ECU=\{E_{1},E_{2},\ldots E_{k},\ldots E_{EN}\}$, where EN indicates the number of ECU in $E_{k}$. Each ECU may has two cores: the computing core and HSM core. The computing core is necessary, and it is responsible for the computing tasks, whereas the HSM core is optional, and only those ECUs that include security-critical tasks have HSMs, as security-critical tasks have to send security-critical messages needing to be security-enhanced (such as adding MAC for integrity protection). HSM is used for security protection, such as key storage, MAC generation, message encryption/decryption, and others [26].

### 3.2. Task Model

Each ACPS application is a directed acyclic graph (DAG) G=<V,E>, where *V* is the set of computing tasks and *E* is the set of messages. Each message indicates the communication relation between two adjacent tasks, if two adjacent tasks assigned to different ECUs, the corresponding message needs to be transmitted on CAN FD; or else, two adjacent tasks can communicate with each other through shared memory. The ACPS includes functions with different security requirements, functions such as steering control and braking control are with high security requirement, functions such as window control and remote door lock control have low security requirement [27]. Thus, we divide the computing tasks into two subgroups based on their level of security requirement, namely, security-critical tasks and non-security-critical tasks [28]. For those security-critical tasks, they can only be assigned to those ECUs with HSM. While for non-security-critical tasks, they can be assigned to any ECUs. The set of computing task in $E_{k}$ is indicated as $Z_{k}$, where $Z_{k}=\left\{T_{k,1},T_{k,2},\ldots T_{k,i},\ldots T_{k,TN_{k}}\right\}$, and $TN_{k}$ indicates the number of tasks in $Z_{k}$. The task set of all ECUs is denoted by *Z*, where $Z={⋃}_{\forall k}Z_{k}$, and TN indicates the total number of tasks in *Z*. $T_{k,i}$ is denoted with a 5-tuple: $T_{k,i}=\left\{P_{k,i},C_{k,i},R_{k,i},D_{k,i},Q_{k,i}\right\}$, which indicate the period (in μs), worst-case execution time (WCET, in μs), worst-case response time (WCRT, in μs), deadline (in μs) and security level, respectively. $Q_{k,i}$ is a binary variable, if $Q_{k,i}$=1, it indicates that $T_{k,i}$ is a security-critical task; otherwise, $T_{k,i}$ is a non-security-critical task. We assume that deadline equals to period for each task, and priorities are assigned to tasks based on their periods, these assumptions are common in ACPS [29,30,31]. $P_{k,i}$, $C_{k,i}$, $D_{k,i}$, and $Q_{k,i}$ are given, and $R_{k,i}$ is calculated as follows:

(1) $$R_{k,i}=C_{k,i}+\sum_{\forall T_{k,i^{\prime }}\in hp_{k,i}}\left\lceil \frac{R_{k,i}}{P_{k,i^{\prime }}}\right\rceil \times C_{k,i^{\prime }}$$

where $hp_{k,i}$ indicates the set of tasks with higher priorities than $T_{k,i}$ in $E_{k}$.

### 3.3. Message Model

Computing tasks of each ACPS functions are allocated to different ECUs, and messages are exchange between different ECUs to realize the communication and cooperation of them, thus CAN FD messages have the same security requirement with their sending tasks. $M_{k}$ indicates the set of messages sent by $ECU_{k}$, where $M_{k}=\left\{m_{k,1},m_{k,2},\ldots m_{k,j},\ldots m_{k,MN_{k}}\right\}$, and $MN_{k}$ indicates the number of messages in $M_{k}$. The message set of all ECUs is denoted by *M*, where $M={⋃}_{\forall k}M_{k}$, and MN indicates the total number of messages in *M*. $m_{k,j}$ is indicated with a 5-tuple: $m_{k,j}=\left\{p_{k,j},c_{k,j},r_{k,j},d_{k,j},q_{k,i}\right\}$, which indicate the period (in μs), worst-case transmission time (WCTT, in μs), WCRT (in μs), deadline(in μs) and security level of $m_{k,j}$, respectively. $p_{k,j}$ and $c_{k,j}$ are given, $q_{k,j}$ equals to that of its sending task, and $d_{k,j}$ equals to $p_{k,j}$ [29,30,31]. As the authors of [32] verified that the rate monotonic priority order is close to the optimal priority order for CAN, thus we assume that the rate monotonic priorities are assigned to CAN FD messages. CAN FD messages are scheduled non-preemptively before transmitting on the bus, thus $r_{k,j}$ is calculated as follows [30,33]:

(2) $$\begin{array}{c} \forall m_{k,j},m_{k^{\prime },j^{\prime }},r_{k,j}=\\ block_{k,j}+c_{k,j}+\sum_{\forall m_{k^{\prime },j^{\prime }}\in shp_{k,j}}\left\lceil \frac{r_{k,j}}{p_{k^{\prime },j^{\prime }}}\right\rceil \times c_{k^{\prime },j^{\prime }} \end{array}$$

where $block_{k,j}$ indicates the blocking delay incurred by low priority messages, $shp_{k,j}$ indicates the set of messages with higher priorities than $m_{k,j}$ in *M*.

As it shows in Figure 1, we try to reduce the security-related hardware cost by minimize the number of HSMs required for a given CAN FD-based ACPS, where both the task mapping, task scheduling and message scheduling are explored subject to end-to-end deadline and security constraints. Thus, we need to define the end-to-end delay for each function path. A function path $PH_{l}$ is an ordered interleaving sequence of tasks and messages, where $PH_{l}=\left[T_{l,1},m_{l,1},T_{l,2},m_{l,2},\ldots ,T_{l,n-1},m_{l,n-1},T_{l,n}\right]$, and *n* indicates the number of tasks in $PH_{l}$. PH indicates the set of function paths in ACPS. We assume an asynchronous sampling communication approach is employed in ACPS, thus the end-to-end WCRT of $PH_{l}$ (we indicate it as $PR_{l}$) can be calculated by adding the WCRT of all computing tasks and CAN FD messages on the path, the security-enhancing related delay overhead, as well as the periods of all the messages and their receiving tasks on the path [34]. To sum up, $PR_{l}$ is calculated as follows:

(3) $$PR_{l}=\sum_{T_{k,i}\in PH_{l}⋀m_{k,j}\in PH_{l}}\left(R_{k,i}+P_{k,i}+r_{k,j}+p_{k,j}+l_{k,j}\times o_{k,j}\right)$$

where $o_{k,j}$ indicates the security-enhancing related delay overhead. As messages are processed with the same operations non-preemptively inside the HSM [35], we assume an equal $o_{k,j}$ for all security-critical messages, and it is set according to the real implementation of [11]. For each function path, there is an end-to-end deadline, and we indicate it as $PD_{l}$. APCS is schedulable and safe only when all their included function paths meet the deadline constraint.

## 4. Hardware Cost Minimization Algorithms

### 4.1. Stepwise Decreasing Based Heuristic Algorithm

Randomized optimization algorithms such as simulated annealing and genetic algorithm are widely employed to solve research problems similar to this paper [24,36]. However, they only evaluate a finite number of design point based on random changes, thus as the design space expands, they are easily trapped into local optimum, and the global optimum can not be assured. To solve this problem, this paper proposes a stepwise decreasing based heuristic algorithm (SDH) based on the basic ideas of SA. The main differences between SDH and SA are as follows: (1) SDH tries to reduce the search space to a smaller one by implementing a stepwise decreasing on the number of HSMs. As the search space is reduced, it is more likely to find a better task assignment result; (2) in each searching loop when the number of HSMs is given, as long as one feasible task assignment result that meets both the end-to-end deadline and security constraint is found, the heuristic searching step is terminated, thus this allows SDH to reduce the number of HSMs quickly. Details of SDH is given in Algorithm 1.

HN indicates the minimal number of HSMs that are required to be attached to ECUs to meet the design constraints of ACPS, $HN^{\prime }$ is a temporary variable used in each searching loop, where both HN and $HN^{\prime }$ are initially set as EN. $PR_{avg}$ and $PD_{avg}$ indicate the average end-to-end WCRT and average end-to-end deadline of all function paths, respectively. flag indicates if the task assignment result can meet the deadline constraint or not, and it is initially set as TRUE. For SDH, there is an outer while loop (from line 3 to line 27) that realize the stepwise reduction on the number of HSMs, where if the inner while loop can get a feasible task assignment meeting both the real-time and security constraint of ACPS (from line 8 to line 26), the number of HSMs can be reduced by 1, and the outer while loop continues; otherwise, the outer while loop is terminated. Inside the outer while loop, an initial task assignment result is obtained by randomly assigning tasks to ECUs (line 4), and then the average end-to-end WCRT is analyzed (line 5). Next, the inner while loop attempts to decrease the end-to-end WCRT of function paths to meet the end-to-end deadline constraint, which heuristically exchanging tasks assigned to two different ECUs or moving tasks from one ECU to another ECU (line 10). After the timing analysis of the updated task assignment result (line 11), if the end-to-end WCRT if reduced, the new task assignment result is accepted; otherwise, accept it with a certain random probability based on the average end-to-end WCRT difference between current task assignment result and the new task assignment result (from line 12 to line 15). If all function paths can meet the deadline constraints, it means that one feasible task assignment result is found, thus $HN^{\prime }$ can be reduced by 1, and the outer while loop continues (from line 16 to line 20); or else, flag is set as FALSE to indicate that current searching step can not find a feasible task assignment result(from line 21 to 23). If the inner while loop cannot find a feasible task assignment result in all the heuristic searching steps, the outer while loop is terminated and HN is returned as the final result.
**Algorithm 1:** Stepwise Decreasing-Based Heuristic Algorithm.**Input:***Z*, *EN* **Output:***EN*
1: *HN* = *EN*, *HN*′ = *EN*;
2: *flag* = *TRUE*;
3: **while** (*HN*′ ≥ 1) ∧ (*flag* = *true*
**do**;
4:  *Result* = Task_Allocation (*Z*, *EN*, *HN*′);
5:  *PR_avg_* = E2EWCRT_Analysis(*Result*);
6:  *T_ini_* = 3 ∗ *EN*, *T_ter_* = 0.5, *step_num* = 5 ∗ *TN* ∗ *MN*, θ = 0.98;
7:  *T* = *T_ini_*;
8:  **while**
*T* = > *T_ter_*
**do**
9:   **for**
*i* = 1 to *step_num*
**do**
10:    *Result*′ = Heuristic_Task_Move (*Result*);
11:    *PR_avg_*′ = E2EWCRT_Analysis (*Result*);
12:    **if** (*PR_avg_*′ < *PR_avg_*) ∨ (*exp*((*PR_avg_* − *PR_avg_*′)/*T* > *Rand* (0, 1)) **then**
13:     *PR_avg_* = *PR_avg_*′;
14:     *Result* = *Result*′
15:    **end if**
16:    **if** (*PR* < *PD*) **then**
17:     *flag* = *TRUE*;
18:     *HN* = *HN*′
19:     *HN*′ = *HN*′ − 1
20:     break;
21:    **else**
22:     *flag* = *FALSE*;
23:    **end if**
24:   **end for**
25:    *T* = *T* ∗ θ;
26:  **end while**
27: **end while**

To sum up, SDH realizes a stepwise decrease of the design space by reducing the number of HSM one by one. For a given system configuration, as long as one viable task assignment solution is found, the searching step is terminated immediately. Thus, SDH can shorten the searching time to find a viable task assignment solution for the given number of HSMs. Furthermore, the proactive reduction of search space taken by SDH makes it easier to find a viable task mapping solution with fewer HSMs.

### 4.2. Interference Balancing Based Heuristic Algorithm

As SDH is implemented based on the basic ideas of SA, the efficiency of it will deteriorate as the search space expands. Moreover, as the number of HSMs decreases, it becomes increasingly difficult for the heuristic searching to find a feasible task assignment, thus the runtime of SDH increases rapidly. To remedy the above mentioned disadvantages, this paper proposes another interference balancing-based heuristic algorithm IBH. As the number of messages that are scheduled and transmitted on CAN FD depends on the task assignment, and tasks contribute a larger part to the end-to-end WCRT than that of the messages, tasks and their WCRT analysis are primarily considered during the DSE process of IBH. To be more specific, IBH employs the definition of variance to describe difference of interferences caused by the set of tasks assigned to different ECUs. The interference variance is defined as Definition 1 shows.

**Definition** **1.** *The interference variance is the interference differences caused by the set of tasks assigned to different ECUs for a given time interval*.

For a given time interval len, the interference variance is calculated as Equation (4) shows.

(4) $$inf\_variance=\sum_{k=1}^{EN}\frac{inf_{k}^{len}-inf_{avg}^{len}}{EN}$$

where $inf_{k}^{len}$ indicates the interference caused by the set of tasks assigned to $E_{k}$, and the $inf_{avg}^{len}$ indicates the average inf of all ECUs. By assigning task to the ECU that causes the minimal interference variance, the interference caused by those already assigned tasks as well as the end-to-end WCRT of the function paths can be balanced. Details of IBH is given in Algorithm 2.
**Algorithm 2:** Interference Balancing-Based Heuristic Algorithm.**Input:***Z*, *EN* **Output:***HN*
1: *HN* = *EN*, *flag* = *TRUE*;
2: Task_Sort (Z);
3: **while** (*HN* ≥ 1) ∧ (*flag* = *TRUE*) **do**
4:   *Interval* = Min_Period (Z;
5:  **for**
*i* = 1 to *TN*
**do**
6:   *Variance* = *W* (1, *TN*);
7:   **if**
*Q_i_* == 1 **then**
8:    **for**
*k* = 1 to *HN*
**do**
9:     Result = Task_Allocation (*T_i_*, *E_k_*);
10:     **if**
*T_i_* is scheduleable **then**
11:      *Variance* (1, *k*) = Variance_Analysis (*Interval*);
12:      **end if**
13:    **end for**
14:   **else**
15:    **for**
*k* = 1 to *EN*
**do**
16:     Result = Task_Allocation (*T_i_*, *E_k_*);
17:     **if**
*T_i_* is schedulable **then**
18:      *Variance* (1, *k*) = Variance_Analysis (*Interval*);
19:     **end if**
20:    **end for**
21:   **end if**
22:   (*Min_Variance*, *k*) = min (*Variance*);
23:   **if**
*Min_Variance* < *W*
**then**
24:    *flag* = *TRUE*;
25:    Allocate *T_i_* to the *E_k_*;
26:    *Interval* = *P_i_*;
27:   **else**
28:    *flag* = *false*;
29:    break;
30:   **end if**
31:  **end for**
32:  if system is schedulable **then**
33:   *HN* = *HN*-1;
34:  **else**
35:   break;
36:  **end if**
37: **end while**

For IBH, *HN* also indicates the minimal number of HSMs that are required to be attached to ECUs to meet the design constraint of ACPS *flag* indicates if each task can be successfully assigned to an ECU where it can meet the deadline constraint, *Interval* indicates the time interval that the WCRT is analyzed for the tasks, and *Variance* is an array to store the interference variances when tasks are assigned to different ECUs. *HN* is initially set as *EN*, and *flag* is initially set as *TRUE*. IBH first sorts task with increasing period and size (line 2), and then there is a while loop tries to find a feasible task assignment result (it means that all function paths meet their end-to-end deadline constraint, and the system is schedulable) when the number of HSMs is set as *HN* (from line 3 to line 37). Inside the while loop, *Interval* is initially set as the minimal task period (line 4), and next there is a for loop tries to assign each task to the ECU with the minimal variance (from line 5 to line 31). Inside the for loop, each element of *Variance* is initially set as a big number *W* (line 6). Moreover, if the current task is a security-critical task, it tries to assign it to the ECUs with HSMs, and the corresponding variance is analyzed(from line 7 to line 14); or else if the current task is a non-security-critical task, it tries to assign it to all ECUs, and the corresponding variance is analyzed(from line 15 to line 21). After the analysis of all possible variances, if the current task can be successfully assigned to ECUs (which means that all assigned tasks are schedulable), it is assigned to the ECU with the minimal variance (from line 22 to line 25), and *Interval* is updated to the period of the current task (line 26); or else, the current task can not be assigned successfully, which means that it can not find a feasible task assignment result when the number of HSMs is set as *HN* (from line 27 to line 30). Thus, the for loop is terminated. After the for loop, if all tasks can be assigned properly, *HN* is reduced by 1, and the while loop continues (from line 32 to line 34); or else, the while loop is terminated (from line 35 to line 36). *HN* is returned as the minimal number of HSMs that are required to meet both the security and real-time constraints.

To sum up, IBH also realizes a stepwise decrease of the design space by reducing the number of HSM one by one, the difference between IBH and SDH is that IBH replaces the random searching approach with interference balancing-based approach to find a viable task assignment for the given system configuration. The interference balancing-based approach is easier to find a schedulable task assignment result, as it considers to balance the interference to the unassigned tasks in each step.

## 5. Experiment Results

We conducted extensive experiments based on both synthetic and real data sets to verify the proposed SDH and IBH algorithm, and the efficiency of the proposed algorithms is shown by comparing them with the simulated annealing-based heuristic algorithm presented in [24]. This algorithm is state-of-the-art, and we indicate it as SSH. The task graph generator given in [37] is used to generate synthetic ACPS functions, where the parameters of the tasks and messages are generated according to the guidelines on real-world automotive benchmarks [38]. For synthetic data sets, the number of ECUs is set as 6/10/14/18, and the number of tasks is set as 80/100/120/140/160/200. Six real-life ACPS functions given in [15] are adopted to generate the real data sets, where the number of tasks are increased by replicating the ACPS functions. For real data sets, the number of ECUs is set as 6/8/10/12, and the number of tasks is set as 40/60/80/100/120. For both synthetic and real data sets, the percent of security-critical tasks is set as 20%/40%/60%. $o_{k,j}$ is set according to the real implementation given in [35], where $o_{k,j}$ = 103 μs. We assume that the arbitration phase bit-rate and data phase bit-rate of CAN FD are 500 kbps and 2 Mbps [30,31], respectively. The experiments are conducted on an OS X(v10.13.1) machine running on 2.3 GHz the 7th generation Intel i5 core with 8 GB main memory. The experiment code is implemented in Matlab 2017a.

**Figure 2:**
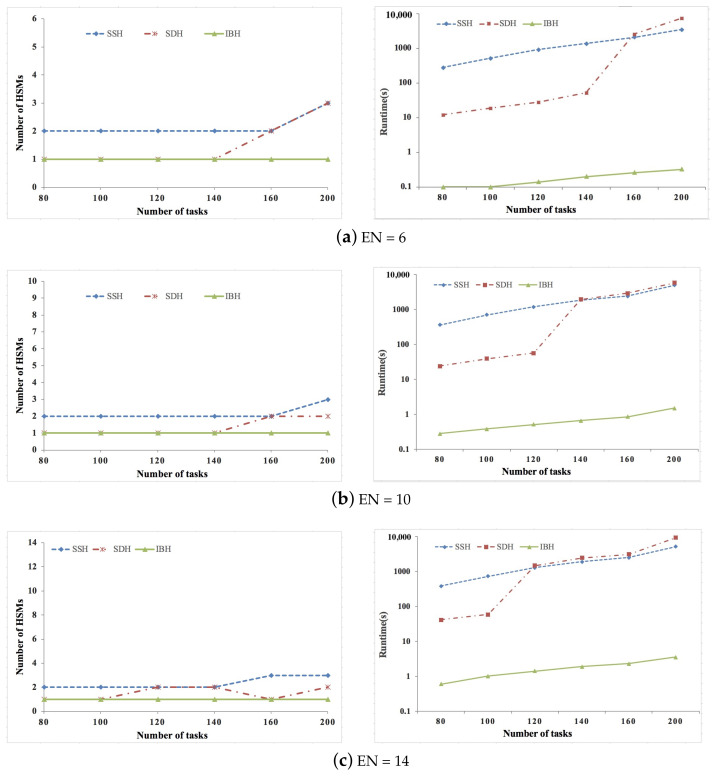

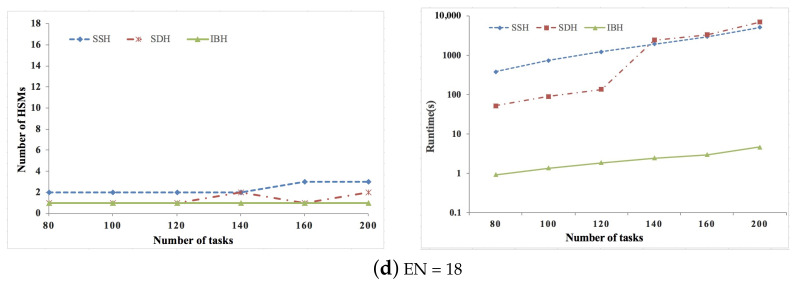
Experimental results of synthetic data set when percent of security-critical task is 20%.

**Figure 3:**
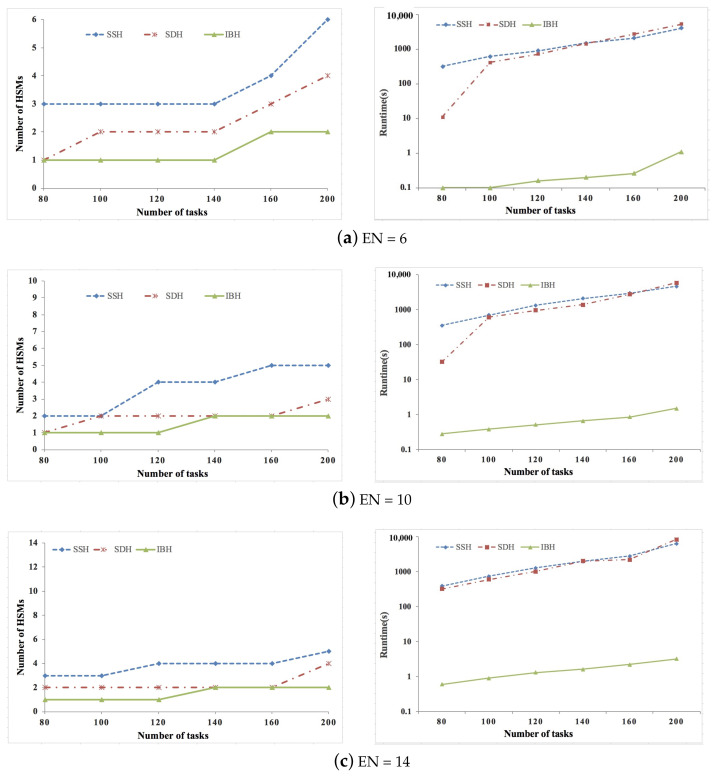

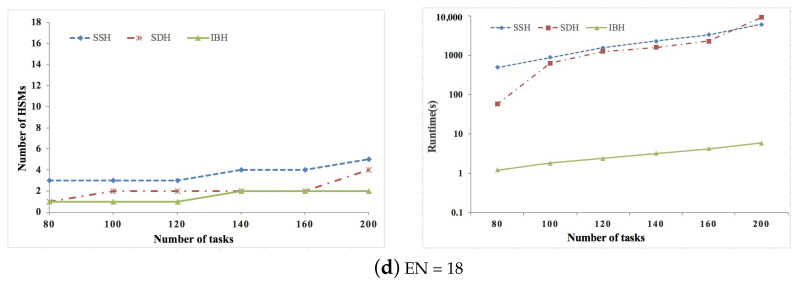
Experimental results of synthetic data set when percent of security-critical task is 40%.

**Figure 4:**
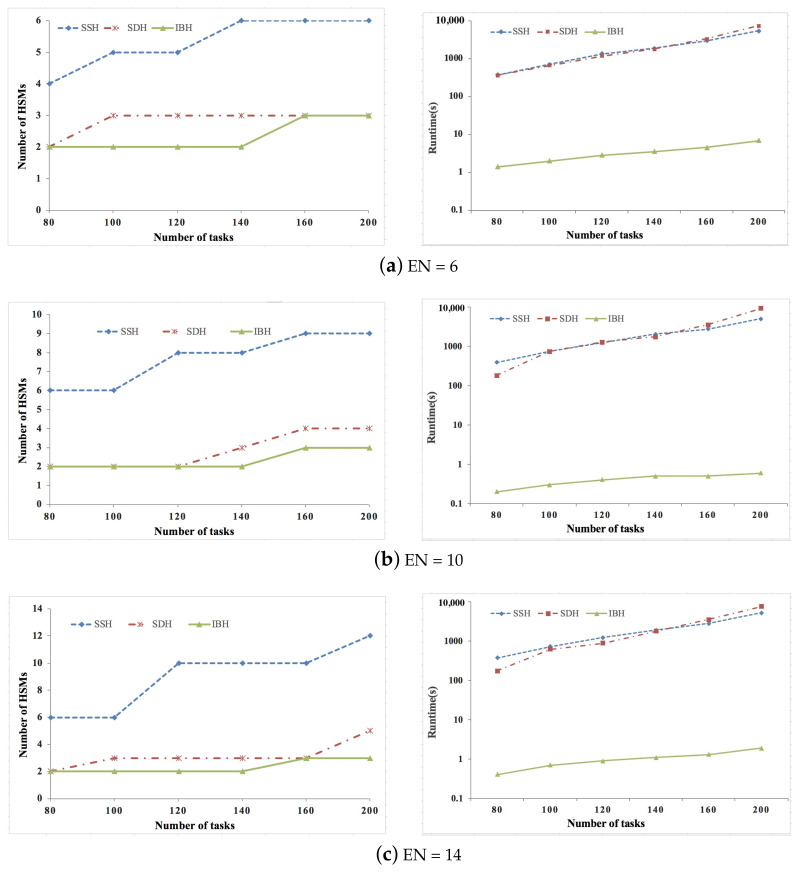

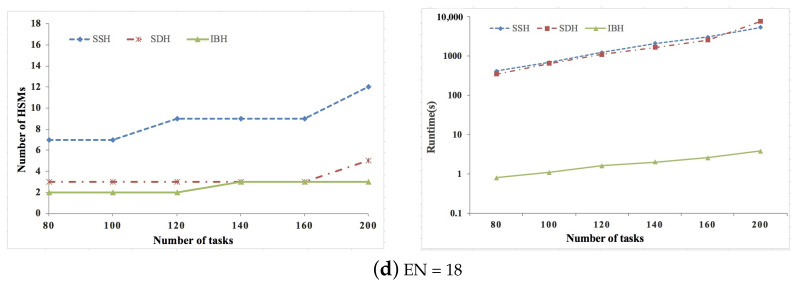
Experimental results of synthetic data set when percent of security-critical task is 60%.

**Figure 5:**
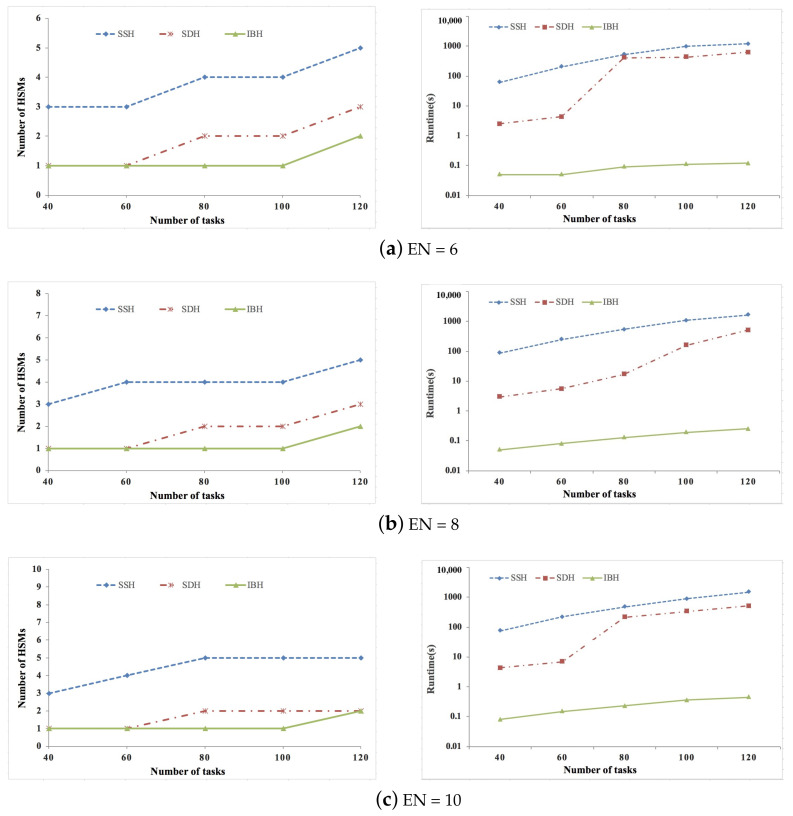

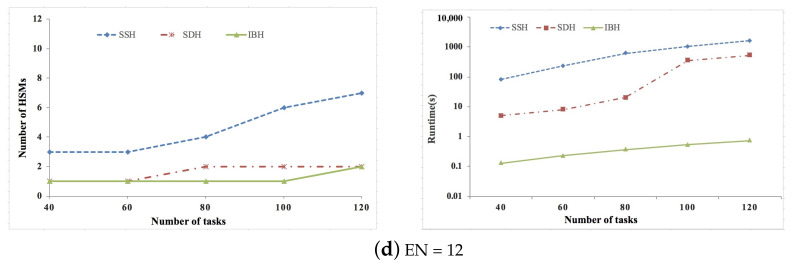
Experimental results of real data set when percent of security-critical task is 20%.

**Figure 6:**
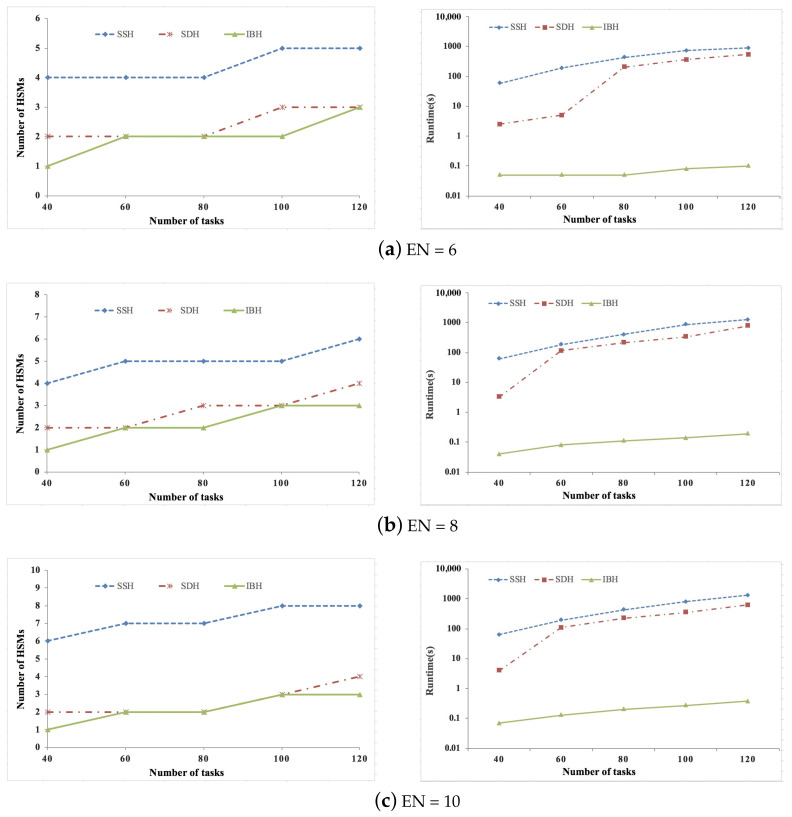

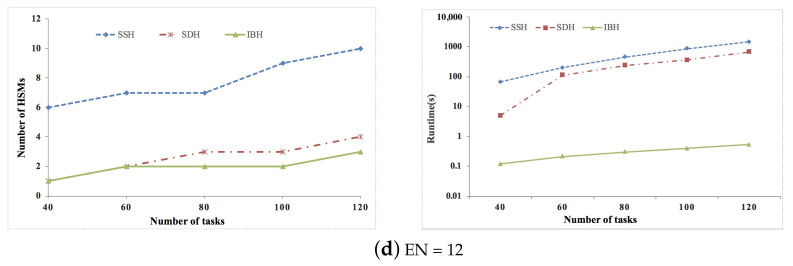
Experimental results of real data set when percent of security-critical task is 40%.

**Figure 7:**
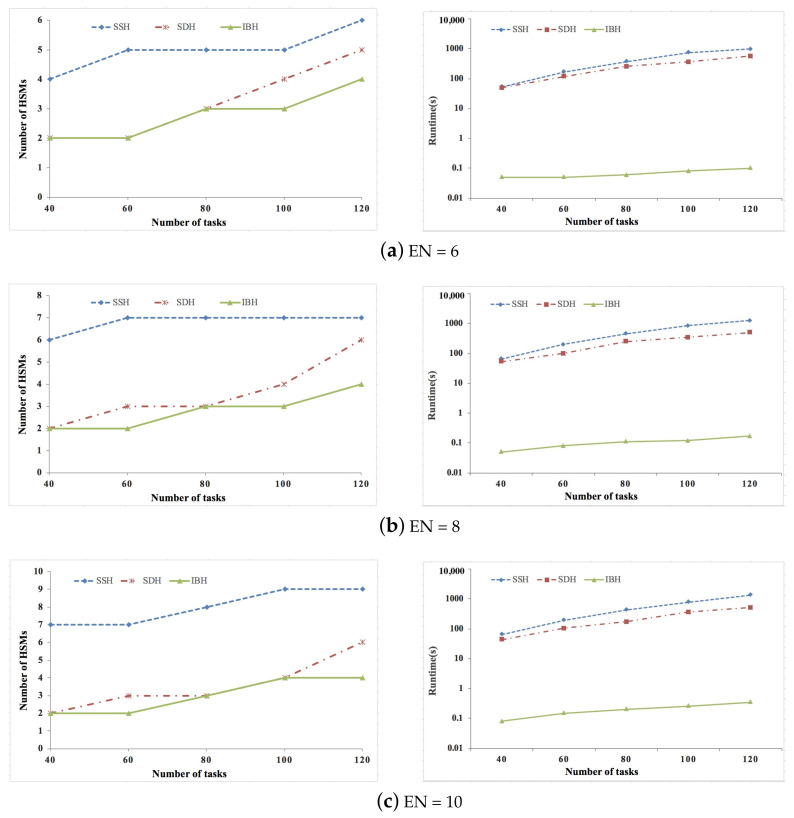

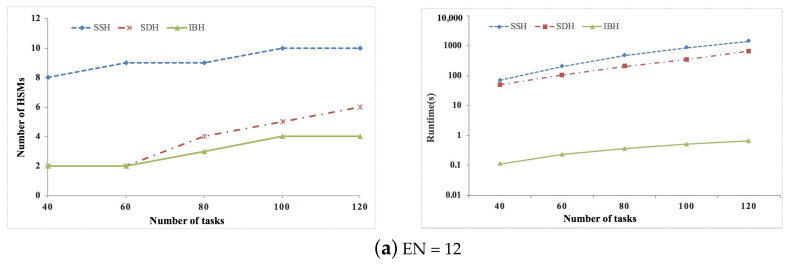
Experimental results of real data set when percent of security-critical task is 60%.

Figure 2, Figure 3 and Figure 4 show the experimental results of synthetic data sets, where the percent of security-critical tasks is set as 20%, 40%, and 60%, respectively. Figure 5, Figure 6 and Figure 7 show the experimental results of real data sets, where the percent of security-critical tasks is also set as 20%, 40%, and 60%, respectively. From the above mentioned results, we can conclude that the proposed SDH and IBH are better than SSH in reducing the security-related hardware cost for ACPS. Especially as the increase about the percent of security-critical tasks, the advantage of SDH and IBH becomes more obvious. For synthetic data sets, the hardware cost can be reduced by 61.4% and 45.6% averagely for IBH and SDH, respectively, and for real data sets, the hardware cost can be reduced by 64.3% and 54.4% averagely for IBH and SDH, respectively. Furthermore, as the expansion of the design space due to the increase of ECUs and tasks, IBH shows a better performance than SDH in reducing the number of HSMs in most cases, and it also gets the same performance with SDH in the other cases. Last but not the least, the runtime of IBH is two or three orders of magnitude smaller than SDH and SSH, which means that SDH is quite extensible.

## 6. Conclusions

As connectivity between and within automobiles increases, it brings great cyber-security challenges for CAN FD-based ACP. The auto industry recommends employing the HSM-based multicore ECUs to secure the ACPS functions with acceptable delay overhead. However, the introduction of HSM incurs significant hardware cost. In this paper, we try to reduce security-enhancing-related hardware cost by proposing two efficient DSE algorithms, namely, SDH and IBH, which explore the task assignment, task scheduling, and message scheduling to minimize the number of required HSMs. Experiments on both synthetical and real data sets show that the proposed SDH and IBH are superior than the state-of-the-art SSH algorithm, and the advantage of SDH and IBH becomes more obvious as the increase about the percentage of security-critical tasks. Furthermore, IBH is better than SDH, and the runtime of IBH is two or three orders of magnitude smaller than SDH and SSH.

## Data Availability

Not applicable.
